# Supplementary material for: Sodium Butyrate Induces Mitophagy and Apoptosis of Bovine Skeletal Muscle Satellite Cells through the Mammalian Target of Rapamycin Signaling Pathway
Source: Int J Mol Sci. 2023 Aug 30;24(17):13474. doi: 10.3390/ijms241713474 (PMC10487490; doi:10.3390/ijms241713474)
Supplement: Supplementary file 1 [file ijms-24-13474-s001.zip › ijms-2540070-supplementary.pdf]

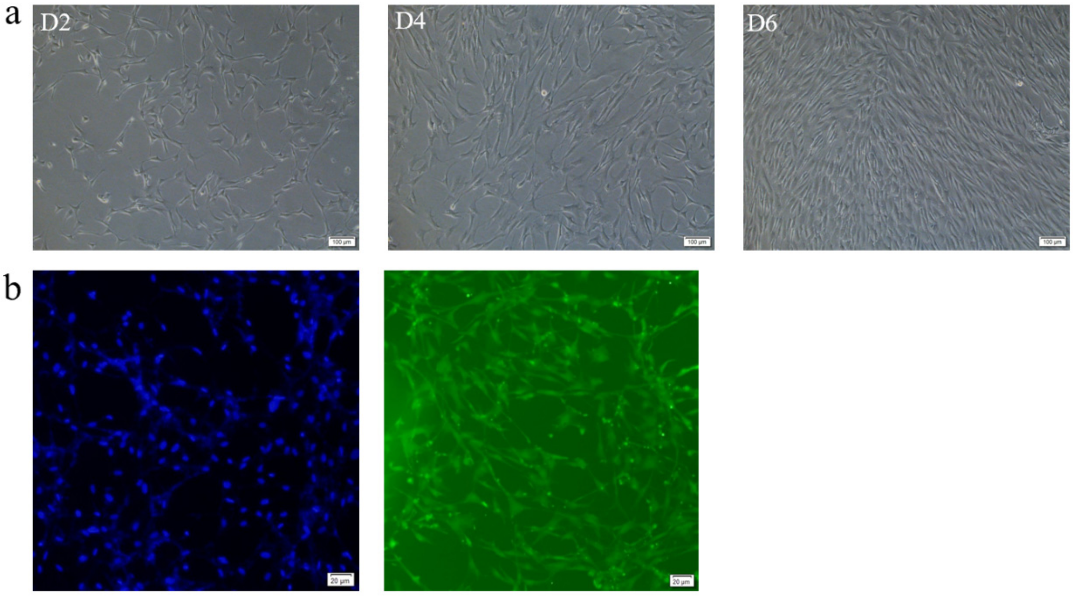

Figure S2. State of BSCs. a: State of BSCs in different proliferation periods; b:Immunofluorescence of BSCs. Left: Nuclear staining results; Right: Desmin antibody immune cell result.
