# Supplementary material for: Sodium Butyrate Induces Mitophagy and Apoptosis of Bovine Skeletal Muscle Satellite Cells through the Mammalian Target of Rapamycin Signaling Pathway
Source: Int J Mol Sci. 2023 Aug 30;24(17):13474. doi: 10.3390/ijms241713474 (PMC10487490; doi:10.3390/ijms241713474)
Supplement: Supplementary file 1 [file ijms-24-13474-s001.zip › Table S1 Primer sequence.pdf]

Table S1. Primer sequence and product length

Gene	Accession number	Primer sequence （5’-3’）	Product length（bp）
*CCNB*2	NM_174264.3	F: ACCAAGTTCACCGCCATCAA<br>R: TGCACGAACGACACACTCTT	160
*CDK*1	NM_174016.2	F:AAGCTGGCGCTTGGAAGTTA<br>R:GTATGGTAGACCCCGGCTTT	193
PCNA	NM_001034494.1	F: GAACCTCACCAGCATGTCCA<br>R: ACGTGTCCGCGTTATCTTCA	219
Bax	NM_173894.1	F:GCAAACTGGTGCTCAAGG<br>R: GCACTCCAGCCACAAAGA	125
Bcl-2	NM_001166486.1	F: TGAGTTCGGAGGGGTCATGT<br>R:GGTAAAAGCTGTGAAAGGATCCAG	143
*p*53	NM_174201.2	F:ACTTGTGGAACCTACTTCCTG<br>R: GAGGGGACAAAGGACGACAG	257
*LC*3*B*	NM_001001169.1	F: GCCGAACCTTCGAACAAAGA<br>R: TTGAGCTGTAAGCGCCTTCT	193
*p*62	NM_001205519.1	F:TTTACTCCGACTGTTAATGCAATC<br>R:TGTTTACCAGACCGTCCAGC	70
*PINK*1	NM_001099701.2	F:GGAACTGGATGCAGATGGCT<br>R:CCCTGGCCGTAAAAGGGATT	261
mTOR	XM_002694043.6	F:CCTTGGCACAACAGTGCATC<br>R:AGGTCCTCATGTCCTCGTGA	285
*EIF*4*EBP*1	NM_001077893.2	F: GGAGTGTCGGAACTCACCTG<br>R: AACTGTGACTCTTCACCGCC	71
*FOXO*1	XM_025000053.1	F:CAAGCGAGCAAGCAGGCTA<br>R:GTCTTCGCTGCCAAGTCTGA	82
*AKT*1	NM_173986.2	F:CCACCTGACCAAGACGACAG<br>R:CCCCTCAGGCCGTGC	188
GAPDH	NM_001034034.2	F:TCGGAGTGAACGGATTCGGC<br>R:ATGGCGACGATGTCCACTTT	82
