# Supplementary figures and images for: Sodium Butyrate Induces Mitophagy and Apoptosis of Bovine Skeletal Muscle Satellite Cells through the Mammalian Target of Rapamycin Signaling Pathway
Source: Int J Mol Sci. 2023 Aug 30;24(17):13474. doi: 10.3390/ijms241713474 (PMC10487490; doi:10.3390/ijms241713474)

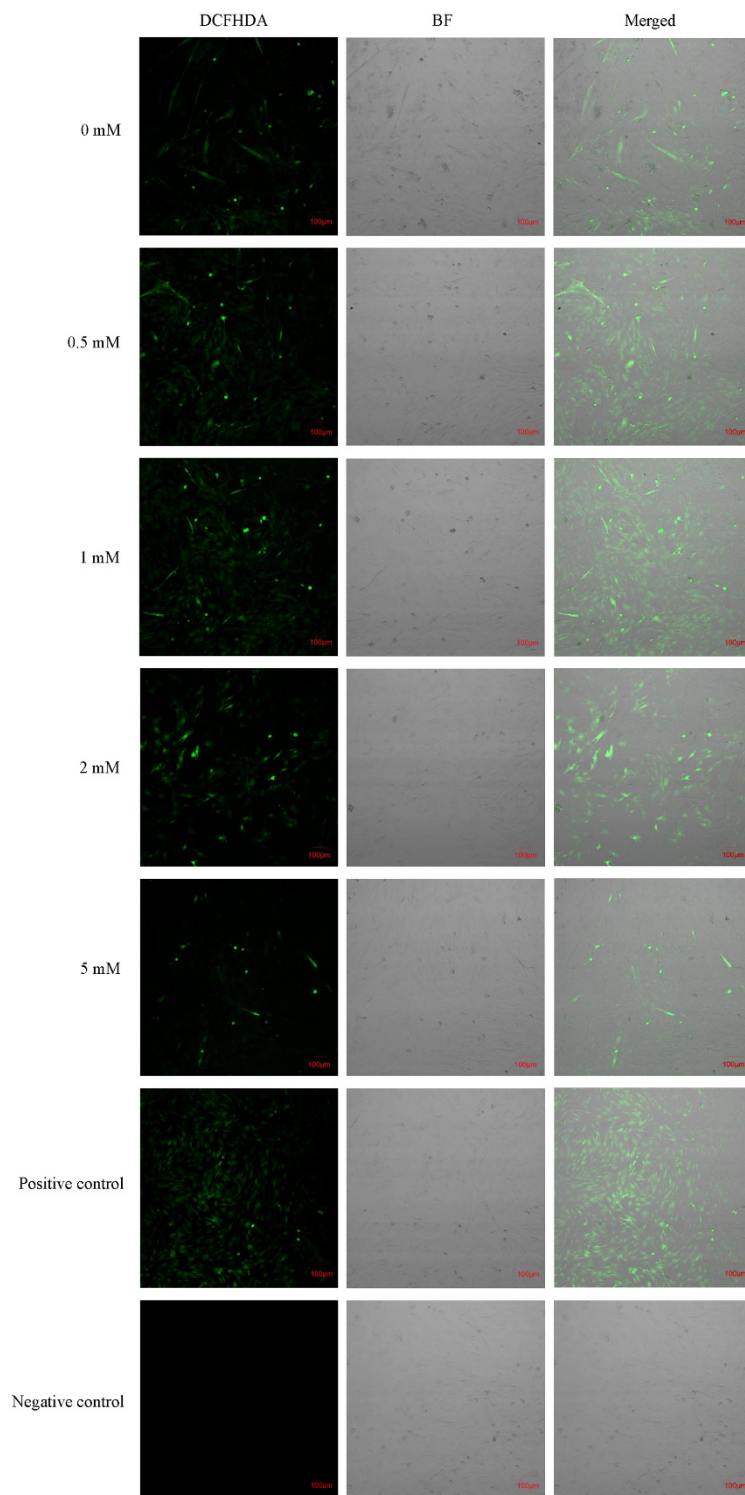

**Figure S1.** Effect of NaB on ROS level

Supplement: Supplementary file 1 [file ijms-24-13474-s001.zip › Figure S1 ROS Level.pdf]
